# From self-regulated learning to learning outcomes in virtual learning environments: motivational activation and flow as a sequential psychological pathway

**DOI:** 10.3389/fpsyg.2026.1898113

**Published:** 2026-08-21

**Authors:** Jin Zhang, Shaofeng Yang, Jinhai Liu, Ke Zhu

**Affiliations:** 1 Faculty of Education, Henan Normal University, Xinxiang, China; 2 School of Mathematics and Statistics, Henan Normal University, Xinxiang, China; 3 Henan Provincial Collaborative Innovation Center for Intelligent Education, Henan Normal University, Xinxiang, China

**Keywords:** flow experience, intrinsic motivation, learning engagement, learning satisfaction, self-regulated learning, sequential mediation, virtual learning environments

## Abstract

**Introduction:**

Virtual learning environments have become an important context for higher education, yet the psychological mechanisms through which students achieve positive learning outcomes remain insufficiently understood. Informed by self-determination theory and flow theory, this study examined a sequential psychological pathway linking self-regulated learning to learning engagement and learning satisfaction through intrinsic motivation and flow experience. By integrating self-regulatory, motivational, and experiential processes within a single model, this study moves beyond research that has examined only direct relationships or treated intrinsic motivation and flow experience as parallel mechanisms.

**Methods:**

A cross-sectional survey was conducted among 531 Chinese university students with experience in virtual learning. Data were analyzed using SPSS 26.0 and SmartPLS 4.0. Partial least squares structural equation modeling and 5,000 bootstrap resamples were used to test the measurement model, direct effects, specific indirect effects, and sequential mediation paths.

**Results:**

Self-regulated learning was positively associated with both learning engagement and learning satisfaction. Intrinsic motivation and flow experience each significantly mediated the relationships between self-regulated learning and the two learning outcomes. More importantly, significant sequential indirect effects were found from self-regulated learning through intrinsic motivation and flow experience to both learning engagement and learning satisfaction. This pattern indicates that higher self-regulated learning was associated with stronger intrinsic motivation, which was further associated with greater flow experience and more positive learning outcomes. The direct effects of self-regulated learning on both outcomes remained significant after the mediators were included, suggesting partial mediation.

**Discussion:**

These findings indicate that positive learning outcomes in virtual learning environments are not only related to learners' self-regulatory capacity but are also associated with motivational activation and immersive learning experiences. By integrating self-regulated learning, intrinsic motivation, and flow experience within a sequential model, the study contributes to educational psychology by showing how self-regulatory capacity is linked to learning outcomes through successive motivational and experiential processes. Practically, this sequential pattern suggests that virtual learning environments should support goal setting, progress monitoring, autonomy, timely feedback, and optimally challenging tasks to promote engagement and satisfaction.

## 1 Introduction

Against the backdrop of the continuing digital transformation of higher education, virtual learning environments (VLEs) have become an important context for university students' course learning (Petchamé et al., 2023). In this study, VLEs refer primarily to technology-supported learning environments, including learning management systems, MOOC platforms, synchronous video classrooms, and university online course platforms, rather than being limited to immersive virtual reality environments (Bond et al., 2021). Compared with traditional face-to-face instruction, VLEs rely on online learning systems, digital course resources, interactive feedback tools, micro-videos, and other digital resources to provide learners with greater temporal flexibility, learning autonomy, and resource accessibility (Liu et al., 2025). Previous studies have suggested that digital resources can enrich the presentation of instructional content and provide learners with more opportunities for knowledge exchange, personalized learning, and interactive feedback (Martin and Bolliger, 2018). However, while VLEs enhance learning flexibility, they may also reduce external supervision and immediate interaction, making learners more vulnerable to distraction, learning isolation, and insufficient sustained engagement (Kuo et al., 2014). Therefore, learning effectiveness in online learning environments does not depend solely on technological platforms or digital resources themselves, but also on whether learners can maintain positive psychological involvement and sustained learning behaviors (Broadbent and Poon, 2015). Accordingly, understanding the formation pathways of students' learning engagement (LE) and learning satisfaction (LS) in VLEs has become an important issue in digital learning research and educational psychology.

Among the many factors that influence learning outcomes in VLEs, self-regulated learning (SRL) is regarded as a key psychological capacity for maintaining learning continuity and learning quality in online learning. SRL emphasizes learners' active efforts to set learning goals, plan learning tasks, monitor learning progress, and regulate learning strategies (Yu, 2023). Because VLEs are typically characterized by high autonomy and relatively low external supervision, learners' ability to manage time effectively, sustain attention, adjust strategies, and maintain effort is directly related to their continued engagement and evaluation of the learning experience (Jeno et al., 2018). Previous studies have shown that SRL is closely associated with LE, learning persistence, and LS in online learning contexts (Esteban-Millat et al., 2014; Xiao and Li, 2021). However, examining only the direct relationship between SRL and learning outcomes is insufficient to fully explain why learners are able to sustain engagement and develop positive learning experiences in VLEs.

To further explain the psychological pathways linking SRL and learning outcomes, this study introduces two key psychological variables: intrinsic motivation (IM) and flow experience (FE). According to self-determination theory (SDT), individuals' IM is closely related to psychological experiences such as autonomy and competence (Ryan and Deci, 2020). In VLEs, learners with higher levels of SRL are generally better able to control their learning process, select appropriate strategies, and cope with learning difficulties, which may be associated with higher levels of IM. Meanwhile, flow theory suggests that individuals are more likely to experience an immersive psychological state when they maintain a high level of concentration and perceive a relative balance between challenges and skills (Yang et al., 2025). Learners with stronger IM are more likely to sustain engagement in learning tasks based on interest and perceived value, which may further be linked to deeper FE.

Although previous studies have separately examined the relationships among SRL, IM, FE, and online learning outcomes, three limitations remain. First, existing studies have often focused on the direct association between SRL and LE or LS, while providing insufficient explanation of the internal psychological processes underlying this relationship (Archibald, 2010). Second, IM and FE have frequently been examined as parallel variables, whereas relatively few studies have tested whether they constitute a sequential pathway from “motivational activation” to “experiential deepening” (Nakamura and Csikszentmihalyi, 2014). Third, online learning research has often used a single learning outcome as the dependent variable, with limited attention to LE and LS as two distinct outcome dimensions (Hoffman and Novak, 2009; Makransky and Petersen, 2019). LE emphasizes learners' behavioral, cognitive, and emotional involvement in the learning process, whereas LS reflects learners' overall evaluation of their learning experience and learning outcomes. Therefore, including both variables can provide a more comprehensive understanding of how positive learning outcomes are formed in VLEs.

Based on the above analysis, this study constructs a sequential mediation model to examine the psychological pathways linking SRL and learning outcomes in VLEs. Specifically, this study investigates whether IM and FE sequentially mediate the relationships between SRL and two learning outcomes: LE and LS. It should be noted that, although this study draws on SDT to explain the relationship between SRL and IM, it does not directly measure basic psychological needs such as autonomy, competence, and relatedness. Therefore, this study does not test the complete mechanism of SDT, but rather applies SDT partially to interpret the relationships among the focal variables. The contributions of this study are threefold. Unlike previous studies that primarily examined direct associations between SRL and online learning outcomes, the present study further investigates the motivational and experiential processes through which these relationships may occur. Accordingly, this study proposes the following research questions:

RQ1: In VLEs, is SRL positively associated with LE and LS?
RQ2: Do IM and FE mediate, both independently and sequentially, the relationship between SRL and learning outcomes?

## 2 Literature review and hypotheses

### 2.1 Self-regulated learning in virtual learning environments

SRL refers to the psychological and behavioral process through which learners actively set learning goals in academic contexts and continuously monitor and regulate their cognitive, motivational, and behavioral processes to achieve these goals (Pintrich, 2000; Zimmerman, 2000). This process typically involves key components such as goal setting, learning planning, metacognitive monitor, strategy adjustment, and effort maintenance (Panadero, 2017). SRL represents not only a set of specific learning strategies but also a dynamic system of self-regulatory capacities (Schunk and Jeffrey, 2018). With the continuing development of digital learning environments, SRL has gradually shifted from an auxiliary learning capacity in traditional classrooms to an important psychological foundation for maintaining learning continuity, learner autonomy, and learning quality in online learning contexts.

In VLEs, the learning process is usually characterized by greater autonomy, flexibility, and relatively lower levels of external supervision, meaning that learners assume greater responsibility for their own learning (Wong et al., 2019). Particularly in contexts where face-to-face learning and online learning alternate or are integrated, students are required to independently set goals, manage time, structure their learning environment, seek help, and conduct self-evaluation. As a result, SRL becomes a key capacity for maintaining learning adaptability and learning continuity (Cheng et al., 2025). Learners therefore need to rely on time management, learning planning, cognitive monitoring, and effort regulation to sustain stability and continuity in the learning process (Broadbent and Poon, 2015; Dabbagh and Kitsantas, 2012). Previous studies have shown that higher levels of SRL are closely associated with better learning persistence, academic performance, and online learning adaptability (Barnard-Brak et al., 2010; Cerezo et al., 2016). Therefore, in VLEs, SRL should not be regarded merely as a collection of learning strategies, but rather as a core psychological capacity that enables learners to achieve autonomous learning, sustained engagement, and positive learning experiences in highly flexible learning environments with weakened external supervision.

### 2.2 Self-regulated learning, learning engagement, and learning satisfaction

To comprehensively examine learning outcomes in VLEs, this study uses LE and LS as two outcome variables. LE refers to learners' behavioral involvement, cognitive effort, and emotional participation during the learning process, reflecting their sustained level of investment in learning activities (Dixson, 2015; Fredricks et al., 2004). LS refers to learners' overall positive evaluation of the learning process, learning experience, and learning outcomes based on their own expectations; it is an important affective indicator of the quality of the learning experience (Alqurashi, 2019; Kuo et al., 2014). Although these two constructs are closely related, LE emphasizes learners' state of involvement during the learning process and can be regarded as a process-oriented outcome, whereas LS emphasizes learners' overall evaluation of the learning experience and can be regarded as an evaluative outcome. Therefore, examining both constructs simultaneously can provide a more comprehensive understanding of how learning outcomes are formed in VLEs. Recent research on Chinese university students has also suggested that LS and LE are important psychological variables for explaining students' academic outcomes and may jointly constitute key pathways in the formation of learning outcomes (Qin et al., 2025).

Previous studies have shown that SRL is closely related to LE. Learners with higher levels of SRL are generally able to actively set learning goals, plan learning tasks, monitor learning progress, and adjust learning strategies (Panadero, 2017). Such active regulatory capacities help learners maintain attention, cognitive investment, and behavioral persistence when facing information complexity, external distractions, and low-supervision contexts in VLEs (Broadbent, 2017). Related online learning research has also found that SRL is significantly and positively associated with learners' sustained behavioral, cognitive, and emotional engagement, as well as with higher levels of LE (Albelbisi and Yusop, 2019; Teng and Zhang, 2020).

SRL is also considered to be closely associated with LS. In highly autonomous VLEs, learners often need to rely on their own learning planning and task management abilities to complete learning activities. Learners with higher levels of SRL are usually better able to control the learning process, cope with learning difficulties, and develop a stronger sense of control and perceived competence during learning (Schunk and Jeffrey, 2018). When learners can effectively manage the learning process and maintain a higher level of learning efficacy, they are more likely to develop positive learning experiences and satisfaction evaluations (Eom and Ashill, 2016; Kuo et al., 2014). Based on this reasoning, the following hypotheses are proposed:

*H1a: SRL is positively associated with LE*.
*H1b: SRL is positively associated with LS*.

### 2.3 The mediating effect of intrinsic motivation

In VLEs, learning activities generally depend more heavily on learners' autonomous planning, sustained engagement, and self-management. Therefore, SRL may be closely related to learners' IM. According to SDT, IM primarily arises from individuals' experiences of autonomy and competence during an activity (Ryan and Deci, 2020). Learners with higher levels of SRL are usually able to actively set learning goals, plan learning tasks, monitor learning progress, and adjust learning strategies (Panadero, 2017). This active regulatory process helps learners develop a sense of control and perceived competence in learning activities, which may in turn be associated with higher levels of IM (Schunk and Jeffrey, 2018). Online learning research has also shown significant positive relationships among SRL, IM, learning persistence, and LE (Albelbisi and Yusop, 2019).

The formation of online LE is influenced not only by external instructional support but also by learners' internal motivational states. Previous research has found that different dimensions of teaching presence, including course design and organization, discourse facilitation, direct instruction, assessment, and technical support, can explain students' behavioral, cognitive, and emotional engagement to varying degrees (Wang, 2022). This suggests that LE in VLEs is not determined solely by the platform environment; whether learners can develop sustained internal motivation is also important. IM is considered a key psychological force that promotes online LE and positive learning experiences. Unlike extrinsic motivation, intrinsically motivated learning behavior is mainly driven by learners' interest in the learning activity itself, value identification, and self-satisfaction (Deci et al., 1991). When learners participate in learning based on internal interest, they are more likely to actively engage in learning tasks and maintain higher levels of persistence when facing cognitive load and learning challenges (Ryan and Deci, 2020). Previous studies have shown that higher levels of IM are closely related to learners' sustained behavioral, cognitive, and emotional engagement and also contribute to more positive learning experiences and satisfaction evaluations (Schunk and DiBenedetto, 2020). Therefore, IM may mediate the relationships between SRL and LE and between SRL and LS.

It should be noted that this study does not directly measure basic psychological needs such as autonomy, competence, and relatedness. Therefore, it does not test the full mechanism of SDT, but rather uses SDT as a theoretical basis to explain the relationship between SRL and IM. Based on this reasoning, the following hypotheses are proposed:

*H2a: IM mediates the relationship between SRL and LE*.
*H2b: IM mediates the relationship between SRL and LS*.

### 2.4 The mediating role of flow experience

FE refers to an optimal psychological state formed when individuals are highly concentrated, deeply involved, and experience a match between challenge and ability during an activity (Csikszentmihalyi, 1990). In VLEs, the formation of flow typically depends on conditions such as clear learning goals, continuous feedback, focused attention, and a balance between challenge and skill (Panadero, 2017). As online learning becomes increasingly autonomous and interactive, FE has gradually been regarded as an important psychological variable for explaining digital LE and positive learning experiences (Bond et al., 2021).

SRL may be closely related to FE. Learners with higher levels of SRL are usually able to actively plan learning tasks, set clear goals, and continuously monitor progress and adjust strategies during the learning process (Panadero, 2017). This regulatory capacity helps learners enhance their sense of control over learning tasks, maintain focused attention and cognitive involvement, and thus become more likely to develop immersive learning experiences (Teng and Zhang, 2020). This is particularly important in VLEs, where learners face challenges such as information complexity, external distractions, and self-paced learning. The goal management, strategy regulation, and attention control involved in SRL may help learners maintain a relative balance between challenge and ability and may therefore be positively associated with FE.

FE is also closely related to LE and LS. Learners in a state of flow usually demonstrate higher levels of concentration, behavioral persistence, and cognitive involvement, making them more likely to sustain LE (Csikszentmihalyi, 1990). Online learning research has shown that FE is significantly associated with LE, continuance intention, and positive learning experiences (Esteban-Millat et al., 2014). In addition, because FE is accompanied by a stronger sense of control and positive emotions, learners are more likely to evaluate the overall learning process positively and report higher levels of LS (Li et al., 2023). Therefore, FE may mediate the relationships between SRL and LE and between SRL and LS. Based on this reasoning, the following hypotheses are proposed:

*H3a: FE mediates the relationship between SRL and LE*.
*H3b: FE mediates the relationship between SRL and LS*.

### 2.5 The sequential mediating effect of intrinsic motivation and flow experience

In addition to their independent mediating roles, IM and FE may operate as sequential psychological processes. Previous studies have highlighted the respective roles of self-regulatory and motivational processes in shaping online learning outcomes (Chiu, 2022; Jansen et al., 2019). More directly, Shi et al. (2025) found that academic self-concept and flow sequentially mediated the relationships between engaging online learning environments and both deep and shallow cognitive engagement, suggesting that personal psychological resources and immersive experiences may function in a progressive sequence. However, that study focused on academic self-concept rather than IM, leaving unclear whether IM and FE form a comparable sequential pathway between SRL and learning outcomes. Integrating SDT and flow theory, the present study proposes that learners with stronger SRL may be better able to experience a sense of control and competence, which may be associated with higher levels of IM. In turn, intrinsically motivated learners may sustain greater concentration and involvement and become more deeply immersed in learning activities, ultimately reporting higher levels of LE and LS.

Integrating SDT and flow theory, SRL may first be associated with learners' IM, while IM may further be linked to higher levels of concentration, involvement, and immersive experience, ultimately relating to LE and LS.

Specifically, in VLEs, SRL emphasizes learners' active efforts to set goals, plan tasks, monitor progress, and regulate strategies (Panadero, 2017). This process helps learners develop a sense of control and perceived competence in learning activities and may therefore be associated with higher levels of IM. Previous studies have shown that learners' self-regulatory capacities are closely related to autonomous motivation and sustained LE (Jeno et al., 2018). Furthermore, when learners participate in learning based on interest, value identification, and internal satisfaction, they are more likely to maintain focused attention and develop deep immersive experiences. Flow theory suggests that flow is essentially an autotelic experience, in which individuals continue to engage in a task because of the satisfaction derived from the activity itself (Nakamura and Csikszentmihalyi, 2014). Online learning research has also found significant associations among autonomous motivation, FE, continuance intention, and LS (Chiu, 2022).

Therefore, IM and FE may jointly constitute a sequential mediation path between SRL and learning outcomes. On the one hand, SRL may enhance learners' IM and encourage them to engage more actively in learning tasks. On the other hand, IM may be further associated with FE, enabling learners to maintain higher levels of concentration, behavioral persistence, and positive emotional experience during virtual learning. Previous studies have shown that FE is significantly related to LE, learning persistence, and LS (Esteban-Millat et al., 2014; Shin, 2006). Based on this reasoning, the following hypotheses are proposed:

*H4a: IM and FE sequentially mediate the relationship between SRL and LE*.
*H4b: IM and FE sequentially mediate the relationship between SRL and LS*.

**Figure 1:**
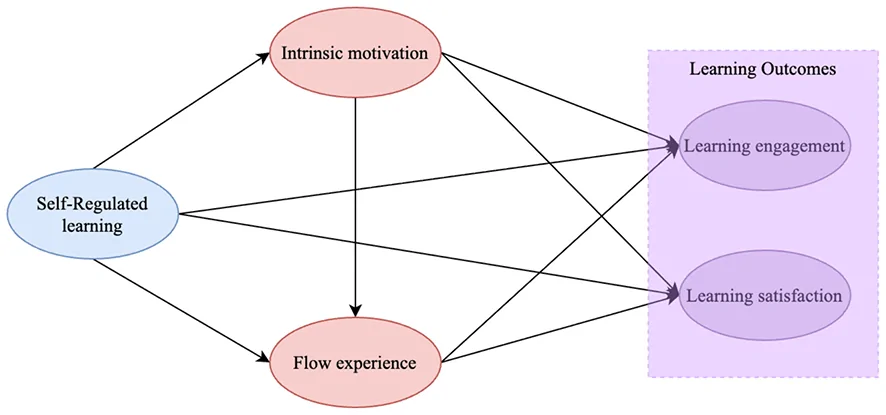
Proposed research model.

In VLEs, learning outcomes are reflected not only in learners' sustained engagement but also in their subjective evaluation of the overall learning experience. LE reflects learners' behavioral, cognitive, and emotional involvement in the learning process and can be regarded as a process-oriented outcome. LS reflects learners' overall evaluation of the learning experience and learning outcomes and can be regarded as an evaluative outcome (Kuo et al., 2014). Therefore, examining both constructs simultaneously helps provide a more comprehensive understanding of how positive learning outcomes are formed in VLEs. Based on the above analysis, the relationship between SRL and learning outcomes may not be limited to direct associations; it may also operate through the sequential psychological pathway of “IM → FE” to influence LE and LS. Accordingly, this study develops an integrated model based on SDT and flow theory to examine the sequential mediating roles of IM and FE in the relationships between SRL and two types of learning outcomes. The proposed research model is shown in Figure 1. Intrinsic motivation is expected to precede flow experience because it provides the internal psychological energy for sustained task involvement (Ryan and Deci, 2020), whereas flow represents an immersive experiential state that generally emerges during continued engagement in an activity (Csikszentmihalyi, 1990). Unlike a parallel mediation model, the proposed sequential model therefore specifies a directional process from motivational activation to experiential immersion.

## 3 Methods

### 3.1 Participants and procedure

This study adopted a cross-sectional survey design to examine the relationships among SRL, IM, FE, LE, and LS among university students with virtual learning experience. Participants were recruited from Chinese universities using convenience sampling. Eligible participants were required to be currently enrolled university students, have prior experience with virtual learning, and voluntarily agree to participate in the study. Convenience sampling was adopted because it enabled efficient access to university students with recent virtual learning experience; however, the resulting sample should not be regarded as fully representative of the wider population of Chinese university students.

In this study, virtual learning referred to course learning conducted through learning management systems, MOOC platforms, synchronous video conferencing tools, or university online course platforms, such as LMSs, MOOCs, Tencent Meeting, Rain Classroom, Xuexitong, Canvas, and Moodle. Before completing the questionnaire, participants were instructed to recall their most recent or most frequently used virtual learning experience and respond to the items based on that experience. The questionnaire was distributed online through Wenjuanxing^1^ and shared via university course groups, learning communities, and social media.

The study was reviewed and approved by the Ethics Committee of the Faculty of Education, Henan Normal University (Approval Code: 2025BS-0616; Approval Date: March 18, 2025). All participants provided informed consent before completing the questionnaire. Participation was voluntary and anonymous, and participants were informed that they could withdraw from the survey at any stage without penalty. No personally identifiable information was collected.

A total of 579 questionnaires were collected. After excluding 48 invalid responses because of abnormal response time, patterned responses, or obvious logical inconsistencies, 531 valid responses were retained, yielding a completion rate of 91.7%. The final sample included 257 males (48.4%) and 274 females (51.6%), and most participants were aged 18–24 years (68.0%). Detailed demographic characteristics are presented in Table 1.

**Table 1:** Demographic characteristics of participants.

Characteristics	Valid sample (*N* = 531)	
	*N*	%
Gender		
Male	257	48.4
Female	274	51.6
Age		
< 18 years	25	4.7
18-20 years	158	29.8
21-24 years	203	38.2
>24 years	145	27.3
Grade		
Freshman	80	15.1
Sophomore	154	29.0
Junior	109	20.5
Senior	188	35.4
Virtual learning time		
< 6 months	116	21.8
6 months−1 year	266	50.1
1-2 years	149	28.1
Weekly virtual learning time		
< 5 h	100	18.8
5-10 h	287	54.0
11-20 h	144	27.1

### 3.2 Instruments

This study used a structured questionnaire to measure SRL, IM, FE, LE, and LS. All scales were adapted from established instruments and semantically adjusted to fit the context of VLEs. To ensure the applicability of the scales in the present research context, the research team first translated and contextualized the original items. Experts in educational psychology and online learning were then invited to evaluate the item content, wording, and contextual appropriateness. Based on expert feedback, the wording of some items was revised to improve clarity and content validity. Before the formal survey, a small-scale pilot test was conducted to examine the comprehensibility and fluency of the questionnaire, and minor wording revisions were made accordingly. All items were rated on a five-point Likert scale ranging from 1 = “strongly disagree” to 5 = “strongly agree.” Higher scores indicated higher levels of the corresponding construct. The full measurement items are provided in the Supplementary material.

#### 3.2.1 Self-regulated learning

The SRL scale was mainly adapted from the Self-Regulation for Learning Online scale developed by Broadbent et al. (2023) with further revisions based on Zimmerman (2000) theory of SRL and the structure of the MSLQ developed by Pintrich et al. (1993) The scale consisted of 10 items covering three dimensions: planning control, with 3 items; metacognitive monitor, with 4 items; and effort to control, with 3 items. The items were adapted to the virtual learning context to reflect learners' goal setting, learning monitoring, and effort maintenance in online courses, learning platforms, and synchronous video-based learning. The overall Cronbach's α coefficient of the scale was 0.749. The Cronbach's α coefficients for planning control, metacognitive monitor, and effort to control were 0.790, 0.842, and 0.774, respectively, indicating acceptable internal consistency.

#### 3.2.2 Intrinsic motivation

The IM scale was mainly adapted from the motivational measurement framework proposed by Gagné et al. (2010) and revised for the virtual learning context. The scale consisted of 5 items measuring the extent to which learners participated in virtual learning based on interest, value identification, and self-satisfaction. A sample item is: “I participate in virtual learning because I am interested in the content itself.” The Cronbach's α coefficient of this scale was 0.860, indicating good internal consistency.

#### 3.2.3 Flow experience

The FE scale was adapted from the EduFlow-2 scale developed by Heutte et al. (2021) and revised for the virtual learning context. The scale consisted of 4 items measuring learners' concentration, immersion, and involvement during virtual learning. A sample item is: “When I learn in a virtual environment, I feel completely immersed in the learning process.” The Cronbach's α coefficient of this scale was 0.827, indicating good internal consistency.

#### 3.2.4 Learning engagement

The LE scale was adapted from He et al. (2025), which examined online LE. The scale consisted of 4 items measuring learners' behavioral involvement, cognitive effort, and emotional participation during virtual learning. A sample item is: “When learning in a virtual environment, I can easily concentrate on learning tasks.” The Cronbach's α coefficient of this scale was 0.848, indicating good internal consistency.

#### 3.2.5 Learning satisfaction

The LS scale was adapted from Zhou et al. (2025), which examined online LS. The scale consisted of 5 items measuring learners' overall evaluation of virtual learning in terms of satisfaction with the learning experience, satisfaction with learning outcomes, and willingness to continue learning. A sample item is: “Compared with traditional courses, I am satisfied with my virtual learning experience.” The Cronbach's α coefficient of this scale was 0.866, indicating good internal consistency.

### 3.3 Data analysis

Data were analyzed using SPSS 26.0 and SmartPLS 4.0. First, SPSS 26.0 was used to conduct descriptive statistics for the demographic characteristics of the sample and to calculate the means, standard deviations, and Pearson correlation coefficients of the study variables. Subsequently, SmartPLS 4.0 was used to conduct partial least squares structural equation modeling (PLS-SEM) to examine the measurement model, structural model, and mediation effects (Zhang et al., 2026). PLS-SEM was selected because the study focused on explaining and predicting multiple endogenous constructs and involved a higher-order construct and several direct, indirect, and sequential mediation paths. This approach permits the simultaneous assessment of the measurement and structural models and supports nonparametric bootstrapping for indirect-effect testing (Hair et al., 2022).

For the assessment of the measurement model, Cronbach's α, composite reliability (CR), average variance extracted (AVE), and outer loadings were used to evaluate reliability and convergent validity. The Fornell–Larcker criterion and the heterotrait–monotrait ratio (HTMT) were used to assess discriminant validity. For the assessment of the structural model, multicollinearity, path coefficients, coefficients of determination (*R*^2^), effect sizes (f^2^), and predictive relevance (Q^2^) were examined to evaluate the explanatory power and predictive ability of the model.

The mediation and sequential mediation effects were tested using nonparametric bootstrapping with 5,000 resamples. An indirect effect was considered statistically significant when its 95% confidence interval did not include zero (Preacher and Hayes, 2008). All statistical tests were two-tailed, and the significance level was set at *p* < 0.05.

## 4 Results

### 4.1 Preliminary analysis

#### 4.1.1 Common method bias

Harman's single-factor test was used to examine potential common method bias. The results of exploratory factor analysis showed that seven factors had eigenvalues greater than 1, and the first factor accounted for 36.895% of the total variance, which was below the recommended threshold of 40% (Podsakoff et al., 2003). These results suggest that common method bias was not a serious concern in this study, and the data were suitable for subsequent analyses.

#### 4.1.2 Descriptive and correlation analysis

Pearson correlation analysis was conducted to examine the relationships among the study variables. As shown in Table 2, PC, MM, EC, IM, FE, LE, and LS were all significantly and positively correlated. Among these variables, the strongest correlation was found between LS and FE (*r* = 0.580, *p* < 0.01). IM was also strongly correlated with LE (*r* = 0.531, *p* < 0.01), and LE was significantly correlated with FE (*r* = 0.550, *p* < 0.01). These results provided preliminary support for the subsequent structural model analysis.

**Table 2:** Descriptive statistic and Pearson correlation.

Variable	*M*	*SD*	1	2	3	4	5	6	7
1 = PC	3.604	0.818	1						
2 = MM	3.569	0.892	0.547^**^	1					
3 = EC	3.511	0.845	0.480^**^	0.526^**^	1				
4 = IM	3.499	0.851	0.424^**^	0.454^**^	0.471^**^	1			
5 = FE	3.452	0.846	0.461^**^	0.465^**^	0.466^**^	0.544^**^	1		
6 = LE	3.490	0.872	0.466^**^	0.453^**^	0.478^**^	0.531^**^	0.550^**^	1	
7 = LS	3.461	0.890	0.471^**^	0.491^**^	0.509^**^	0.525^**^	0.580^**^	0.529^**^	1

^*^p < 0.05; ^**^p < 0.01; ^***^p < 0.001. PC, planning control; MM, metacognitive monitor; EC, Efforts to control; IM, Intrinsic Motivation; FE, Flow Experience; LE, Learning Engagement; LS, Learning Satisfaction.

### 4.2 Assessment of measurement model

SmartPLS 4.0 was used to assess the measurement model, including indicator reliability, internal consistency reliability, convergent validity, and discriminant validity. Because SRL was modeled as a second-order construct, the measurement model was assessed in two stages.

First, the first-order measurement model was evaluated. As shown in Table 3, the outer loadings of all measurement items were above 0.70, indicating good indicator reliability. The CR values of all constructs were above 0.70, and the AVE values were above 0.50, suggesting that the first-order measurement model had good internal consistency reliability and convergent validity (Hair et al., 2022).

**Table 3:** The reliability and convergence validity of the measurement model in the first stage.

Constructs	Significance test of parameters				CR	AVE
	Indicator	Loading	*t*	*p*		
EC	EC1	0.821	52.067	^***^	0.869	0.688
	EC2	0.835	57.551	^***^		
	EC3	0.832	56.280	^***^		
FE	FE1	0.831	56.444	^***^	0.885	0.659
	FE2	0.782	36.121	^***^		
	FE3	0.826	59.780	^***^		
	FE4	0.807	47.508	^***^		
IM	IM1	0.778	41.645	^***^	0.899	0.641
	IM2	0.820	56.710	^***^		
	IM3	0.833	61.413	^***^		
	IM4	0.795	45.003	^***^		
	IM5	0.775	34.822	^***^		
LE	LE 1	0.842	64.372	^***^	0.898	0.687
	LE 2	0.842	58.445	^***^		
	LE 3	0.815	49.788	^***^		
	LE 4	0.816	53.610	^***^		
LS	LS 1	0.794	45.637	^***^	0.903	0.651
	LS 2	0.828	57.110	^***^		
	LS 3	0.798	48.719	^***^		
	LS 4	0.795	38.761	^***^		
	LS 5	0.818	55.635	^***^		
MM	MM 1	0.828	59.305	^***^	0.894	0.679
	MM 2	0.822	56.747	^***^		
	MM 3	0.837	56.600	^***^		
	MM 4	0.809	46.702	^***^		
PC	PC 1	0.829	49.696	^***^	0.877	0.704
	PC 2	0.837	52.042	^***^		
	PC 3	0.851	68.319	^***^		

^*^p < 0.05; ^**^p < 0.01; ^***^p < 0.001. PC, planning control; MM, metacognitive monitor; EC, Efforts to control; IM, Intrinsic motivation; FE, Flow experience; LE, Learning engagement; LS, Learning satisfaction; AVE, average variance extracted.

Discriminant validity was assessed using the Fornell–Larcker criterion and the heterotrait–monotrait ratio (HTMT). As shown in Table 4, the square root of the AVE for each first-order latent variable was greater than its correlations with other latent variables, satisfying the Fornell–Larcker criterion (Fornell and Larcker, 1981).

**Table 4:** Fornell–Larcker Criterion.

Constructs	EC	FE	IM	LE	LS	MM	PC
EC	**0.830**						
FE	0.454	**0.812**					
IM	0.469	0.541	**0.800**				
LE	0.460	0.543	0.532	**0.829**			
LS	0.484	0.575	0.530	0.531	**0.807**		
MM	0.525	0.460	0.456	0.446	0.489	**0.824**	
PC	0.444	0.443	0.410	0.436	0.466	0.533	**0.839**

Values of diagonal represent the square root of AVE. PC, planning control; MM, metacognitive monitor; EC, Efforts to control; IM, Intrinsic Motivation; FE, Flow Experience; LE, Learning Engagement; LS, Learning Satisfaction. Bold diagonal values represent the square root of AVE.

The HTMT results are presented in Table 5. The HTMT values among the constructs ranged from 0.528 to 0.676, all of which were below the recommended threshold of 0.85, indicating good discriminant validity (Henseler et al., 2015).

**Table 5:** HTMT discriminant validity assessment.

Constructs	EC	FE	IM	LE	LS	MM	PC
EC							
FE	0.566						
IM	0.574	0.641					
LE	0.567	0.647	0.621				
LS	0.590	0.676	0.612	0.619			
MM	0.649	0.551	0.535	0.528	0.572		
PC	0.567	0.547	0.497	0.532	0.562	0.651	

EC, Efforts to control; FE, Flow Experience; IM, Intrinsic Motivation; LE, Learning Engagement; LS, Learning Satisfaction; MM, metacognitive monitor; PC, planning control.

Second, the measurement model including the second-order construct of SRL was evaluated. As shown in Table 6, the loadings of the three first-order dimensions under the second-order construct of SRL were all significant. Specifically, the loadings of planning control, metacognitive monitor, and effort to control were 0.798, 0.842, and 0.809, respectively. The CR and AVE values of SRL were 0.857 and 0.666, respectively, both meeting the recommended criteria. These results indicate that the second-order construct had good internal consistency reliability and convergent validity (Hair et al., 2022; Sarstedt et al., 2019).

**Table 6:** The reliability and convergence validity of the measurement model in the second stage.

Constructs	Significance test of parameters				CR	AVE
	Indicator	Loading	*t*	*p*		
SRL	EC	0.809	44.828	^***^	0.857	0.666
	MM	0.842	59.885	^***^		
	PC	0.798	43.266	^***^		
FE	FE 1	0.831	58.685	^***^	0.885	0.659
	FE 2	0.782	38.545	^***^		
	FE 3	0.826	57.708	^***^		
	FE 4	0.807	45.744	^***^		
IM	IM 1	0.778	41.894	^***^	0.899	0.641
	IM 2	0.820	58.412	^***^		
	IM 3	0.833	61.346	^***^		
	IM 4	0.795	45.099	^***^		
	IM 5	0.775	34.250	^***^		
LE	LE 1	0.839	61.518	^***^	0.898	0.687
	LE 2	0.844	64.522	^***^		
	LE 3	0.815	51.666	^***^		
	LE 4	0.816	51.339	^***^		
LS	LS 1	0.794	46.733	^***^	0.903	0.651
	LS 2	0.829	61.134	^***^		
	LS 3	0.799	49.748	^***^		
	LS 4	0.795	41.947	^***^		
	LS 5	0.816	53.988	^***^		

^*^p < 0.05; ^**^p < 0.01; ^***^p < 0.001. SRL, Self-Regulated Learning; PC, planning control; MM, metacognitive monitor; EC, Efforts to control; IM, Intrinsic Motivation; FE, Flow Experience; LE, Learning Engagement; LS, Learning Satisfaction; AVE, average variance extracted.

As shown in Table 7, the square root of the AVE for each latent variable was greater than its correlations with other latent variables, further supporting the discriminant validity of the final measurement model.

**Table 7:** Fornell–Larcker Criterion.

Constructs	FE	IM	LE	LS	SRL
FE	**0.812**				
IM	0.541	**0.800**			
LE	0.543	0.531	**0.829**		
LS	0.576	0.530	0.531	**0.807**	
SRL	0.554	0.545	0.548	0.588	**0.816**

Values of diagonal represent the square root of AVE. SRL, Self-regulated learning; IM, Intrinsic Motivation; FE, Flow Experience; LE, Learning Engagement; LS, Learning Satisfaction. Bold diagonal values represent the square root of AVE.

The HTMT results shown in Table 8 also indicated that the HTMT values ranged from 0.612 to 0.728, all below the recommended threshold of 0.85 (Henseler et al., 2015). Therefore, the measurement model demonstrated acceptable reliability, convergent validity, and discriminant validity and was suitable for subsequent structural model analysis.

**Table 8:** HTMT discriminant validity assessment.

Constructs	FE	IM	LE	LS	SRL
FE					
IM	0.641				
LE	0.647	0.621			
LS	0.676	0.612	0.619		
SRL	0.703	0.678	0.687	0.728	

SRL, Self-regulated learning; IM, Intrinsic Motivation; FE, Flow Experience; LE, Learning Engagement; LS, Learning Satisfaction.

### 4.3 Hypothesis testing

#### 4.3.1 Structural model assessment

The structural model was evaluated in terms of multicollinearity, explanatory power, predictive relevance, and path coefficients. First, multicollinearity among the latent variables was assessed. The results showed that the variance inflation factor (VIF) values of the predictor variables ranged from 1.000 to 1.645, all below the recommended threshold of 3.3, indicating that serious multicollinearity was not present in the model (Hair et al., 2022).

The explanatory power and predictive relevance of the structural model are shown in Table 9. The *R*^2^ values for IM, FE, LE, and LS were 0.297, 0.388, 0.419, and 0.460, respectively, indicating that the model had acceptable explanatory power for the endogenous variables. The Q^2^ values for these variables were 0.188, 0.252, 0.283, and 0.295, respectively, all greater than 0, suggesting that the model had good predictive relevance (Hair et al., 2022).

**Table 9:** R2 and Q2 assessment of the structural model.

Constructs	*R* ^2^	*Q* ^2^
IM	0.297	0.188
FE	0.388	0.252
LE	0.419	0.283
LS	0.460	0.295

IM, Intrinsic motivation; FE, Flow experience; LE, Learning engagement; LS, Learning satisfaction.

**Figure 2:**
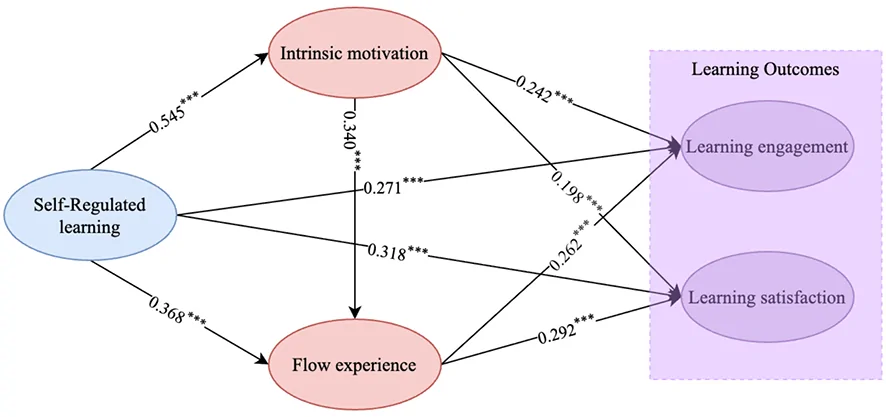
Results of hypothesis testing. ***p < 0.001.

The path analysis results are presented in Table 10 and Figure 2. SRL was significantly and positively associated with IM (β = 0.545, t = 15.278, *p* < 0.001) and FE (β = 0.368, *t* = 7.352, *p* < 0.001). In addition, SRL was significantly and positively associated with LE (β = 0.271, *t* = 4.924, *p* < 0.001) and LS (β = 0.318, *t* = 5.716, *p* < 0.001). Therefore, H1a and H1b were supported.

**Table 10:** Assessment of the structural model.

Paths	Significance Test of Parameters				95% CI		Conclusion	f^2^
	Estimate	STHEV	*t*	*p*			
					Lower	Upper		
FE → LE	0.262	0.053	4.949	^***^	0.159	0.364	Support	0.072
FE → LS	0.292	0.051	5.714	^***^	0.185	0.391	Support	0.097
IM → FE	0.340	0.051	6.725	^***^	0.238	0.439	Support	0.133
IM → LE	0.242	0.053	4.526	^***^	0.135	0.343	Support	0.062
IM → LS	0.198	0.048	4.122	^***^	0.104	0.290	Support	0.045
SRL → FE	0.368	0.050	7.352	^***^	0.271	0.468	Support	0.156
SRL → IM	0.545	0.036	15.278	^***^	0.474	0.613	Support	0.423
SRL → LE	0.271	0.055	4.924	^***^	0.167	0.381	Support	0.077
SRL → LS	0.318	0.056	5.716	^***^	0.214	0.431	Support	0.114

^*^p < 0.05; ^**^p < 0.01; ^***^p < 0.001. STDEV, standard deviation; 95% CI, 95% confidence intervals.

Furthermore, IM was significantly and positively associated with FE (β = 0.340, *t* = 6.725, *p* < 0.001), LE (β = 0.242, *t* = 4.526, *p* < 0.001), and learning satisfaction (β = 0.198, *t* = 4.122, *p* < 0.001). FE was also significantly and positively associated with LE (β = 0.262, *t* = 4.949, *p* < 0.001) and learning satisfaction (β = 0.292, *t* = 5.714, *p* < 0.001). Overall, all direct paths in the structural model reached statistical significance.

#### 4.3.2 Mediating effect analysis

The mediation effects and sequential mediation effects were tested using the bootstrap method with 5,000 resamples. The results are shown in Table 11. First, the total effects were significant. SRL had a significant total effect on LE [β = 0.548, *t* = 14.981, 95% CI (0.469, 0.619)] and learning satisfaction [β = 0.588, *t* = 16.438, 95% CI (0.517, 0.657)].

**Table 11:** Mediating effect analysis.

Paths	Significance test of parameters				95% CI	
	Original sample	STDEV	t	p		
					Lower	Upper
Total effect						
SRL → LE	0.548	0.037	14.981	^***^	0.469	0.619
SRL → LS	0.588	0.036	16.438	^***^	0.517	0.657
Specific indirect effect						
SRL → FE → LE	0.097	0.022	4.454	^***^	0.056	0.142
SRL → FE → LS	0.108	0.021	5.039	^***^	0.066	0.152
SRL → IM → FE	0.186	0.028	6.640	^***^	0.130	0.241
SRL → IM → LE	0.132	0.030	4.444	^***^	0.074	0.191
SRL → IM → LS	0.108	0.027	4.033	^***^	0.056	0.160
IM → FE → LE	0.089	0.024	3.671	^***^	0.046	0.142
IM → FE → LS	0.100	0.025	3.970	^***^	0.053	0.152
SRL → IM → FE → LE	0.049	0.013	3.755	^***^	0.025	0.076
SRL → IM → FE → LS	0.054	0.013	4.080	^***^	0.030	0.082
Direct effect						
SRL → LE	0.271	0.055	4.924	^***^	0.167	0.381
SRL → LS	0.318	0.056	5.716	^***^	0.214	0.431

^*^p < 0.05; ^**^p < 0.01; ^***^p < 0.001. STDEV, standard deviation; 95% CI, 95% confidence interval.

Regarding the specific indirect effects, IM significantly mediated the relationship between SRL and LE [β = 0.132, *t* = 4.444, 95% CI (0.074, 0.191)]. IM also significantly mediated the relationship between SRL and learning satisfaction [β = 0.108, t = 4.033, 95% CI (0.056, 0.160)]. Thus, H2a and H2b were supported.

FE also played a significant mediating role. The indirect effect of SRL on LE through FE was significant [β = 0.097, *t* = 4.454, 95% CI (0.056, 0.142)]. Similarly, the indirect effect of SRL on learning satisfaction through FE was significant [β = 0.108, *t* = 5.039, 95% CI (0.066, 0.152)]. Therefore, H3a and H3b were supported.

The sequential mediation analysis further showed that SRL was indirectly associated with LE through the sequential path of IM and FE [β = 0.049, *t* = 3.755, 95% CI (0.025, 0.076)]. SRL was also indirectly associated with learning satisfaction through the same sequential path [β = 0.054, t = 4.080, 95% CI (0.030, 0.082)]. Therefore, H4a and H4b were supported.

After IM and FE were included in the model, the direct path from SRL to LE remained significant [β = 0.271, t = 4.924, 95% CI (0.167, 0.381)], as did the direct path from SRL to learning satisfaction [β = 0.318, t = 5.716, 95% CI (0.214, 0.431)]. These findings indicate that IM and FE partially mediated the relationships between SRL and the two learning outcomes. Overall, the results supported the proposed sequential mediation model, suggesting that SRL is indirectly associated with LE and learning satisfaction through IM and FE.

## 5 Discussion

### 5.1 Discussion of major findings

Drawing on SDT and flow theory, this study examined the relationships between SRL, LE, and learning satisfaction in VLEs, and further tested the mediating and sequential mediating roles of IM and FE. The results showed that SRL was significantly and positively associated with both LE and learning satisfaction. IM and FE each mediated the relationships between SRL and the two learning outcomes. Moreover, the sequential mediation paths from SRL through IM and FE to LE and learning satisfaction was also supported. These findings suggest that positive learning outcomes in VLEs are related not only to technological platforms and course resources, but also to learners' self-regulatory capacity, internal motivational states, and immersive learning experiences (Dabbagh and Kitsantas, 2012; Panadero, 2017).

First, regarding RQ1, this study found that SRL was significantly and positively associated with both LE and learning satisfaction. According to SRL theory, learners manage their learning process through goal setting, learning planning, process monitoring, and strategy adjustment (Zimmerman, 2002). In VLEs, immediate teacher supervision and external structural support are relatively reduced, meaning that learners need to rely more heavily on time management, metacognitive monitoring, and effort regulation to sustain learning continuity (Broadbent and Poon, 2015; Wong et al., 2019). Therefore, learners with higher self-regulatory capacity are more likely to maintain focused attention, cognitive investment, and behavioral persistence, thereby showing higher levels of LE (Pellas, 2014). At the same time, SRL was also closely related to learning satisfaction. Learners who can effectively manage their learning progress, cope with learning difficulties, and perceive learning progress are more likely to form positive evaluations of their learning experience (Liaw and Huang, 2013). This finding is consistent with previous online learning research, which has identified SRL as an important correlate of online course satisfaction (Kuo et al., 2014; Zheng and Xiao, 2024). Compared with previous studies, the present study simultaneously included LE and learning satisfaction, further suggesting that SRL is related both to sustained involvement in the learning process and to learners' positive evaluation of the overall learning experience (Eom and Ashill, 2016; Fredricks et al., 2004). Compared with traditional face-to-face classrooms, external regulation provided by lecturers, peers, and classroom norms is relatively reduced in VLEs. As a result, learners depend more heavily on their own self-regulatory abilities to manage learning and evaluate their learning experiences. This contextual difference may partly explain why SRL is more closely associated with learning satisfaction in virtual learning environments. This contextual difference may help explain why the association between SRL and learning satisfaction is especially salient in VLEs.

Second, regarding RQ2, this study found that IM significantly mediated the relationships between SRL and the two learning outcomes. According to SDT, IM is closely related to learners' experiences of autonomy and competence during an activity (Ryan and Deci, 2020). In VLEs, learners with stronger self-regulatory capacity are generally better able to select learning strategies, control their learning progress, and cope with learning challenges. These abilities may help them develop a stronger sense of control and perceived competence, which may in turn be associated with higher levels of IM (Cho and Heron, 2015). Previous studies have shown that autonomous motivation is consistently related to LE, academic performance, and positive psychological outcomes (Chiu, 2022; Howard et al., 2021). The present study further suggests that the relationship between SRL and learning outcomes is not limited to observable learning management processes, but may also operate through learners' internal motivational states. It should be noted that this study did not directly measure basic psychological needs such as autonomy, competence, and relatedness. Therefore, the use of SDT in this study should be understood as a partial theoretical explanation rather than a direct test of the full SDT mechanism.

Third, FE also significantly mediated the relationships between SRL and LE and learning satisfaction. FE refers to a state in which individuals are highly concentrated, deeply involved, and experience a relative balance between task challenge and personal ability (Csikszentmihalyi, 1990). Although VLEs provide greater flexibility and autonomy, they may also create challenges such as distraction, blurred task boundaries, and loss of control over learning pace. Learners with higher levels of SRL are more capable of setting clear goals, adjusting learning strategies, and maintaining attention, which may make them more likely to experience immersion during learning (Esteban-Millat et al., 2014). Previous studies have also indicated that FE in online learning is closely related to perceived control, focused attention, positive emotions, and continuance intention (Heutte et al., 2021). By positioning FE between SRL and learning outcomes, this study further suggests that flow is not merely an incidental emotional state during learning, but an important psychological channel linking learning regulation capacity with positive learning outcomes (Rodríguez-Ardura and Meseguer-Artola, 2016).

Finally, this study supported the sequential mediation paths from SRL through IM and FE to LE and learning satisfaction. Theoretically, these pathways propose that the goal-directed and monitoring processes involved in SRL may create conditions conducive to perceived control, competence, and intrinsic interest. IM may then sustain voluntary task involvement and concentration, facilitating FE, which is subsequently associated with LE and learning satisfaction. The meta-analysis by Theobald (2021) showed that SRL training can improve university students' learning strategies, motivation, and academic performance. Yang et al. (2025) also found that SRL and FE are important factors explaining LE in self-directed e-learning. Compared with previous research, the main contribution of the present study lies in integrating SRL, IM, and FE into a sequential psychological pathway rather than treating them as independent predictors. In this way, the study provides a more integrative psychological framework for understanding the formation of LE and learning satisfaction in VLEs. Accordingly, the findings should be interpreted as offering a partial SDT-based explanation of the observed relationships rather than as validating the complete theoretical mechanism of SDT.

### 5.2 Theoretical and practical implications

This study has three main theoretical implications. First, it extends the explanatory scope of SRL in VLEs. Previous research has often regarded SRL as a learning management capacity involving goal setting, process monitoring, and strategy adjustment (Zimmerman, 2002). The present study further shows that SRL is not only related to the use of learning strategies, but also systematically associated with learners' IM, FE, and positive learning outcomes. This finding helps to conceptualize SRL not merely as a “learning strategy variable,” but as a core psychological resource in VLEs. Second, this study integrates SDT and flow theory into a single analytical framework, revealing a sequential pathway through which SRL is associated with learning outcomes via IM and FE. This finding suggests that learning outcomes in VLEs cannot be explained solely from the perspective of behavioral management; they also require attention to learners' motivational activation and experiential deepening (Ryan and Deci, 2020). Accordingly, this study provides an integrative psychological pathway of “behavioral regulation–motivational activation–experiential deepening” for explaining positive learning outcomes in VLEs. Third, this study included both LE and learning satisfaction as outcome variables, thereby broadening the explanatory dimensions of learning outcomes in VLEs. LE emphasizes learners' behavioral, cognitive, and emotional involvement in the learning process, whereas learning satisfaction emphasizes learners' overall evaluation of their learning experience and learning outcomes (Fredricks et al., 2004). Examining both constructs simultaneously helps provide a more comprehensive understanding of learners' process-oriented involvement and evaluative experience in VLEs.

The findings also have practical implications. First, virtual learning platforms and course designers should provide support for learners' SRL. Platforms can offer tools for goal setting, learning planning, progress visualization, self-monitoring, and learning reflection, together with accessible navigation and appropriately timed learning reminders. These platform functions should be complemented by explicit SRL training, adaptive scaffolding, and differentiated support for learners with varying regulatory abilities. Second, instructors and platforms should support IM by offering meaningful choices, encouraging self-directed goal setting, and allowing appropriate flexibility in learning pace and task completion. Feedback should be timely, specific, and personalized, addressing learners' progress and strategy use rather than focusing only on final performance. Such feedback may strengthen perceived competence, sustain IM, and help learners adjust ineffective learning strategies. Third, virtual course design should emphasize the conditions that facilitate FE. UI/UX design should provide coherent navigation, clear progress indicators, and learning interfaces with minimal distractions. Course content should be organized through progressive scaffolding so that task difficulty remains appropriately aligned with learners' developing skills. Automated support, such as adaptive prompts, targeted feedback, and learning reminders, may offer timely assistance when students encounter difficulties. These features may help learners maintain concentration and develop immersive learning experiences. Finally, platforms and teachers should not rely solely on course completion rates, login frequency, or grades as indicators of learning effectiveness. They should also attend to students' LE and learning satisfaction. Together, these design and instructional practices may support sustained engagement while creating the motivational and experiential conditions associated with flow and positive evaluations of virtual learning.

### 5.3 Limitations and future research

This study has several limitations. First, it adopted a cross-sectional survey design, which limits the ability to infer causal relationships among the variables. Although the study tested mediation and sequential mediation paths based on a theoretical model, the findings should be interpreted as statistical associations rather than causal mechanisms in a strict sense. Future research could use longitudinal designs, experimental designs, or cross-lagged models to further examine the dynamic relationships among SRL, IM, FE, and learning outcomes. Second, this study mainly relied on self-reported questionnaire data, which may be affected by common method bias and subjective response tendencies. Although the results of Harman's single-factor test indicated that common method bias was unlikely to pose a serious threat to the findings, future studies could combine questionnaire data with learning analytics, platform behavior logs, classroom interaction records, or interview data to obtain more comprehensive and objective evidence. Third, although this study used SDT to explain the relationship between SRL and IM, it did not directly measure basic psychological needs such as autonomy, competence, and relatedness. Therefore, this study cannot fully test the “basic psychological need satisfaction–motivation–learning outcomes” pathway proposed by SDT. Future research could further include basic psychological need satisfaction variables to more systematically examine the relationships among SRL, psychological need satisfaction, IM, and learning outcomes. Fourth, the sample of this study consisted of Chinese university students, and the generalizability of the findings to different cultural contexts, educational stages, and learning platforms requires further validation. Previous research has suggested that digital technology integration and its learning-related outcomes are often context-dependent, and that the fit between technological tools and specific instructional tasks may influence the depth and effectiveness of digital learning practices (Liu and Wei, 2026). Therefore, future research could expand the sample sources, compare the stability of the model across different countries, educational levels, and disciplinary backgrounds, and further examine whether platform functions, learning task characteristics, and technology–task fit moderate the relationships among SRL, IM, FE, and learning outcomes. In addition, because all data were collected through online self-reported questionnaires, participants' responses may have been influenced by recall bias, social desirability bias, and common method variance. Although procedural remedies were implemented to reduce these potential biases, future studies are encouraged to employ multiple data sources, probability sampling, or longitudinal designs to further strengthen the robustness and generalizability of the findings.

## 6 Conclusion

This study examined whether IM and FE help explain the associations between SRL and learning engagement and satisfaction in VLEs. Analysis of responses from 531 Chinese university students showed that SRL was positively associated with both outcomes. IM and FE each accounted for significant indirect associations, and the proposed sequential pathway was supported while the direct associations remained significant. These findings extend direct-effect accounts of SRL by positioning self-regulation, intrinsic motivation, and flow as connected components of virtual learning. Practically, the results highlight the value of combining goal-setting and progress-monitoring tools with autonomy-supportive feedback, scaffolded course content, and appropriately challenging tasks. Together, the study provides a more integrated account of how self-regulatory, motivational, and experiential factors are associated with engagement and satisfaction in VLEs.

## Data Availability

The data analyzed in this study is subject to the following licenses/restrictions: The data used to support the findings of this study are available from the corresponding author upon request. Requests to access these datasets should be directed to Jinhai Liu.

## Footnotes

1
https://www.wjx.cn/
