# Supplementary material for: From self-regulated learning to learning outcomes in virtual learning environments: motivational activation and flow as a sequential psychological pathway
Source: Front Psychol. 2026 Aug 21;17:1898113. doi: 10.3389/fpsyg.2026.1898113 (PMC13541931; doi:10.3389/fpsyg.2026.1898113)
Supplement: Supplementary file 1 [file Table_1.DOCX]

**Virtual learning environment learning questionnaire**

| **Constructs** | **English version** | **Chinese version** | **Sources** |
| --- | --- | --- | --- |
| Self-Regulated Learning（SRL） |  |  | Zimmerman (2000); Broadbent et al. (2023) |
| Planning Control （PC） | PC1. In virtual learning, I will set clear learning goals for myself. | PC1.在虚拟学习中，我会为自己设定明确的学习目标。 |  |
|  | PC2.I will plan the completion time of the virtual learning task in advance. | PC2.我会提前规划虚拟学习任务的完成时间。 |  |
|  | PC3.Before I start learning, I will arrange the learning steps. | PC3.在开始学习前，我会安排好学习步骤。 |  |
| Metacognitive Monitor（MM） | MM1. In the process of virtual learning, I will continue to check whether I understand the learning content. | MM1.在虚拟学习过程中，我会持续检查自己是否理解学习内容。 | Pintrich et al. (1991); Broadbent et al. (2023) |
|  | MM2.When I find that I do not understand, I will adjust the learning method. | MM2.当我发现自己不理解时，会调整学习方法。 |  |
|  | MM3.I will reflect on whether my learning effect has reached my expectations. | MM3.我会反思自己的学习效果是否达到预期。 |  |
|  | MM4.I will adjust my learning strategies according to my learning progress. | MM4.我会根据学习进展调整学习策略。 |  |
| Efforts to Control(EC) | EC1.Even if online learning content is difficult, I will continue to complete it. | EC1.即使在线学习内容困难，我也会坚持完成。 | Pintrich et al. (1991); Broadbent et al. (2023) |
|  | EC2.In the learning process, I can stay focused and not easily distracted. | EC2.在学习过程中，我能保持专注，不易分心。 |  |
|  | EC3.I can overcome the interference and continue online learning. | EC3.我能够克服干扰，持续进行在线学习。 |  |
| Intrinsic Motivation(IM) | IM1.I participate in virtual learning because I am interested in learning content. | IM1.我参与虚拟学习是因为我本身对学习内容感到兴趣。 | Gagné et al. (2010) |
|  | IM2.Even if there is no external reward, I will take the initiative to engage in online learning. | IM2.即使没有外在奖励，我也会主动投入在线学习。 |  |
|  | IM3.When the learning content is challenging, I feel motivated to overcome it. | IM3.当学习内容具有挑战性时，我会感觉很有动力去克服它。 |  |
|  | IM4.I enjoy the process of exploring and mastering new knowledge in virtual learning. | IM4.我在虚拟学习中享受探索和掌握新知识的过程。 |  |
|  | IM5.I think the virtual learning process itself is attractive, not just for performance. | IM5.我认为虚拟学习过程本身具有吸引力，而不只是为了成绩。 |  |
| Flow Experience(FE) | FE1.I can easily focus on learning tasks in a virtual learning environment. | FE1.我在虚拟学习环境中很容易专注于学习任务。 | Heutte et al. (2021) |
|  | FE2.When I study in a virtual environment, I feel completely immersed in the learning process. | FE2.当我在虚拟环境学习时，我感到完全沉浸在学习过程中。 |  |
|  | FE3.When learning in a virtual environment, I often feel that time passes quickly. | FE3.虚拟环境学习时，我常觉得时间过得很快。 |  |
|  | FE4.I feel that the challenge of content in virtual learning just matches my ability. | FE4.我感觉虚拟学习中内容的挑战正好匹配我的能力。 |  |
| Learning Engagement(LE) | LE1.I am fully engaged in virtual learning tasks and is not easily disturbed. | LE1.我在虚拟学习任务中全身心投入，不容易被干扰。 | He et al. (2025) |
|  | LE2.I am willing to take the initiative to participate in interaction and discussion in virtual learning. | LE2.我在虚拟学习时愿意主动参加互动与讨论。 |  |
|  | LE3.In the face of difficult tasks, I am still willing to stick to the completion of learning tasks. | LE3.面对困难任务时，我仍然愿意坚持完成学习任务。 |  |
|  | LE4.Virtual learning environment makes me feel excited and engaged. | LE4.虚拟学习环境中令我感到兴奋和投入。 |  |
| Learning Satisfaction.(LS) | LS1.I am satisfied with my overall learning experience in the virtual learning environment. | LS1.我对自己在虚拟学习环境中的整体学习体验感到满意。 | Zhou,T.,Chen,Y.,Li,X., Yang, L., & Zhao, Z. (2025). |
|  | LS2.The resources and support provided by the virtual learning course make me satisfied. | LS2.虚拟学习课程提供的资源和支持让我感到满意。 |  |
|  | LS3.I am satisfied with my academic performance and progress in the virtual environment. | LS3.我对自己的在虚拟环境中的学习成绩和进步感到满意。 |  |
|  | LS4.Compared with traditional learning, I feel good about the experience of virtual learning. | LS4.与传统学习相比，我对虚拟学习的体验感到良好。 |  |
|  | LS5.I am willing to continue to participate in future virtual learning courses. | LS5.我愿意继续参与未来的虚拟学习课程。 |  |

**Reference**

Gagné, M., Forest, J., Gilbert, M.-H., Aubé, C., Morin, E., & Malorni, A. (2010). The Motivation at Work Scale: Validation Evidence in Two Languages. *Educational and Psychological Measurement*, *70*(4), 628-646. <https://doi.org/10.1177/0013164409355698>

Zhou, T., Chen, Y., Li, X., Yang, L., & Zhao, Z. (2025). The relationship between online learning self-efficacy and learning engagement: the mediating role of achievement motivation and flow among registered nurses [Original Research]. *Frontiers in Psychology*, *Volume 16 - 2025*. <https://doi.org/10.3389/fpsyg.2025.1629174>

Broadbent, J., Panadero, E., Lodge, J. M., & Fuller-Tyszkiewicz, M. (2023). The self-regulation for learning online (SRL-O) questionnaire. *Metacognition and Learning*, *18*(1), 135-163. <https://doi.org/10.1007/s11409-022-09319-6>

Zimmerman, B. J. (2000). Attaining self-regulation: A social cognitive perspective. *In M. Boekaerts, P. R. Pintrich, & M. Zeidner (Eds.), Handbook of self-regulation (pp. 13–39). Academic Press.*

Pintrich, P. R., Smith, D. A. F., Garcia, T., & Mckeachie, W. J. (1993). Reliability and Predictive Validity of the Motivated Strategies for Learning Questionnaire (Mslq). *Educational and Psychological Measurement*, *53*(3), 801-813. <https://doi.org/10.1177/0013164493053003024>

Heutte, J., Fenouillet, F., Martin-Krumm, C., Gute, G., Raes, A., Gute, D., Bachelet, R., & Csikszentmihalyi, M. (2021). Optimal Experience in Adult Learning: Conception and Validation of the Flow in Education Scale (EduFlow-2) [Original Research]. *Frontiers in Psychology*, *Volume 12 - 2021*. <https://doi.org/10.3389/fpsyg.2021.828027>

He, J., Wang, Q., & Lee, H. (2025). Enhancing online learning engagement: teacher support, psychological needs satisfaction and interaction. *BMC Psychology*, *13*(1), 696. <https://doi.org/10.1186/s40359-025-03016-0>
